# *Pdgfra* and *Pdgfrb* Genetically Interact in the Murine Neural Crest Cell Lineage to Regulate Migration and Proliferation

**DOI:** 10.3389/fphys.2020.588901

**Published:** 2020-11-02

**Authors:** Julia Mo, Robert Long, Katherine A. Fantauzzo

**Affiliations:** Department of Craniofacial Biology, School of Dental Medicine, University of Colorado Anschutz Medical Campus, Aurora, CO, United States

**Keywords:** Pdgfra, Pdgfrb, neural crest, craniofacial, migration, proliferation

## Abstract

Cranial neural crest cells (cNCCs) are migratory, multipotent cells that originate from the forebrain to the hindbrain and eventually give rise to the cartilage and bone of the frontonasal skeleton, among other derivatives. Signaling through the two members of the platelet-derived growth factor receptor (PDGFR) family of receptor tyrosine kinases, alpha and beta, plays critical roles in the cNCC lineage to regulate craniofacial development during murine embryogenesis. Further, the PDGFRs have been shown to genetically interact during murine craniofacial development at mid-to-late gestation. Here, we examined the effect of ablating both *Pdgfra* and *Pdgfrb* in the murine NCC lineage on earlier craniofacial development and determined the cellular mechanisms by which the observed phenotypes arose. Our results confirm a genetic interaction between the two receptors in this lineage, as phenotypes observed in an allelic series of mutant embryos often worsened with the addition of conditional alleles. The defects observed here appear to stem from aberrant cNCC migration, as well as decreased proliferation of the facial mesenchyme upon combined decreases in PDGFRα and PDGFRβ signaling. Importantly, we found that PDGFRα plays a predominant role in cNCC migration whereas PDGFRβ primarily contributes to proliferation of the facial mesenchyme past mid-gestation. Our findings provide insight into the distinct mechanisms by which PDGFRα and PDGFRβ signaling regulate cNCC activity and subsequent craniofacial development in the mouse embryo.

## Introduction

The various populations of neural crest cells (NCCs) within the vertebrate embryo play critical roles in development and contribute to a wide array of derivatives. In mammals, these cells originate at the neural ectoderm border and undergo an epithelial-to-mesenchymal transition before delaminating from the cranial neural folds or dorsal neural tube. Cranial NCCs (cNCCs) are a subpopulation of NCCs that arise from the forebrain to the hindbrain and eventually contribute to the cartilage and bone of the frontonasal skeleton, as well as the cartilages of the jaw, middle ear, hyoid, and thyroid, among other derivatives (Trainor, 2005; Mayor and Theveneau, 2013). Craniofacial development in the mouse begins around embryonic day (E) 9.5 with the formation of five facial prominences populated by post-migratory cNCCs. These prominences include the frontonasal prominence and pairs of maxillary prominences (MxPs) and mandibular prominences (MdPs). The frontonasal prominence is divided into the lateral and medial nasal processes (MNPs) upon formation of the nasal pits. These nasal processes will eventually fuse to form the nostrils. An additional fusion event occurs between the MNPs and the MxPs resulting in formation of the upper lip. Concurrently, the secondary palatal shelves appear as outgrowths from the oral surface of the MxPs. The shelves grow downward from the MxPs and subsequently elevate to a horizontal position above the tongue. The palatal shelves grow toward one another and eventually fuse, generating a continuous palate that divides the nasal and oral cavities (Bush and Jiang, 2012). The complex morphogenetic process of craniofacial development requires a precise interplay of multiple cell and tissue types. As such, craniofacial development defects, such as cleft lip and palate, are among the most common birth defects in humans (Parker et al., 2010).

Signaling through the platelet-derived growth factor receptor (PDGFR) family of receptor tyrosine kinases plays a critical role in human craniofacial development. In mammals, there are four PDGF ligands, PDGF-A-D, which interact with two receptors, PDGFRα and PDGFRβ. The homodimers PDGF-AA and PDGF-CC solely activate PDGFRα signaling during mammalian development (Boström et al., 1996; Soriano, 1997; Ding et al., 2004), while the homodimer PDGF-BB exclusively activates PDGFRβ signaling (Levéen et al., 1994; Soriano, 1994). Ligand binding induces PDGFR dimerization and activation of tyrosine kinase domains in the cytoplasmic portion of the receptors. These domains in turn autophosphorylate cytoplasmic tyrosine residues, which are then bound by signaling molecules to activate various intracellular signaling pathways and effect downstream cellular responses (Heldin and Westermark, 1999). In humans, nonsyndromic cleft palate is associated with heterozygous missense mutations in the coding region of *PDGFRA* and single base-pair substitutions in the 3' untranslated region (Rattanasopha et al., 2012). Further, cleft lip and palate are associated with single-nucleotide polymorphisms in the regulatory region of *PDGFC* which reduce transcriptional activity of the promoter (Choi et al., 2009). Alternatively, heterozygous missense mutations in *PDGFRB* have been shown to cause Kosaki overgrowth syndrome (OMIM 616592) and Penttinen syndrome (OMIM 601812), the clinical features of which include facial dysmorphology (Johnston et al., 2015; Takenouchi et al., 2015).

The roles of PDGFRα and PDGFRβ in human craniofacial development are evolutionarily conserved in the mouse. *Pdgfra*-null mouse embryos die at mid-gestation and display facial clefting, subepidermal blebbing, hemorrhaging, edema, defects in the cardiac outflow tract, abnormal neural tube development, mispatterned somites, and extensive skeletal defects affecting cNCC derivatives in the frontonasal skeleton and non-NCC-derived axial skeletal elements (Soriano, 1997). Embryos lacking both *Pdgfa* and *Pdgfc* phenocopy the defects in *Pdgfra-*null embryos (Ding et al., 2004). *Pdgfra* is expressed in migrating cNCCs and in the cNCC-derived facial process mesenchyme during mid-gestation, among other sites, while the ligands *Pdgfa* and *Pdgfc* are expressed in the adjacent epithelium (Morrison-Graham et al., 1992; Orr-Urtreger and Lonai, 1992; Ding et al., 2000; Hamilton et al., 2003; He and Soriano, 2013; Fantauzzo and Soriano, 2016). Embryos in which *Pdgfra* has been conditionally ablated in the NCC lineage using the *Wnt1-Cre* driver (Danielian et al., 1998) exhibit a subset of the phenotypes found in null embryos, such as facial clefting, midline hemorrhaging, defects in the aortic arch, and thymus hypoplasia (Tallquist and Soriano, 2003; He and Soriano, 2013). These *Pdgfra^fl/fl^*;*Wnt1-Cre^+/Tg^* embryos display delayed NCC migration into the frontonasal prominence at E9.5 and fewer NCCs in pharyngeal arches 3–6 at E10.5, with bifurcation of the streams entering these arches in a subset of embryos (He and Soriano, 2013). Additionally, these embryos have decreased proliferation in the frontonasal and medial nasal processes at E9.5 and E11.5, respectively (He and Soriano, 2013). Similarly, PDGFRα signaling regulates cell survival and proliferation of the cNCC-derived mesenchyme of the secondary palatal shelves at E13.5 (Fantauzzo and Soriano, 2014). Conditional ablation of *Pdgfra* specifically in cNCCs using the *Sox10ER^T2^CreER^T2^* driver and following administration of tamoxifen at E7.5 likewise leads to fewer NCCs in the craniofacial region at E10.5, decreased proliferation in the MNP at E11.5, and eventual frontonasal dysplasia (He and Soriano, 2015). Interestingly, use of this driver revealed a novel requirement for PDGFRα in the mandible, as *Pdgfra^fl/fl^*; *Sox10ER^T2^CreER^T2^* embryos additionally exhibited decreased proliferation in the mandibular mesenchyme at E11.5 and mandibular hypoplasia at E16.5 (He and Soriano, 2015). Conversely, *Pdgfrb-* and *Pdgfb-*null mice die near birth and display hemorrhaging, edema, defects in the cardiac ventricular septum, kidney defects, thrombocytopenia, and anemia (Levéen et al., 1994; Soriano, 1994). *Pdgfrb*, like *Pdgfra*, is expressed in the craniofacial mesenchyme during embryogenesis (Soriano, 1994; Fantauzzo and Soriano, 2016; McCarthy et al., 2016) and conditional ablation of *Pdgfrb* in the NCC lineage results in a wider nasal septum (NS), delayed palatal shelf development, and facial subepidermal blebbing in a subset of embryos (Fantauzzo and Soriano, 2016). Though the etiology of these defects is currently unknown, *Pdgfrb^fl/fl^*;*Wnt1-Cre^+/Tg^* embryos do not have obvious defects in cNCC migration at E8.5–E10.5 (Fantauzzo and Soriano, 2016).

The PDGFRs have been shown to genetically interact during murine craniofacial and heart development. A previous report analyzing the effect of simultaneously conditionally ablating *Pdgfra* and *Pdgfrb* in the NCC lineage observed that skeletal preparations of these double-homozygous mutant embryos did not have more severe frontonasal midline defects than those found in *Pdgfra^fl/fl^*;*Wnt1-Cre^+/Tg^* embryos (McCarthy et al., 2016). However, malformations in bones at other locations at E17.5, including the basisphenoid, alisphenoid, and hyoid bones, as well as defects in various cardiac NCC derivatives at E14.5–E18.5, were observed that were more severe than those found in either *Pdgfra* or *Pdgfrb* single-homozygous mutant embryos (Richarte et al., 2007; McCarthy et al., 2016). The latter phenotype was shown to arise from cardiac NCC migration defects into the outflow tract as early as E10.5 and not from defects in proliferation nor survival of cells in the conotruncal region between E10.5 and E12.5 (Richarte et al., 2007). Phosphatidylinositol 3-kinase (PI3K) has been identified as the main downstream effector of PDGFRα signaling during embryonic development in the mouse (Klinghoffer et al., 2002). Embryos homozygous for a constitutive autophosphorylation mutant knock-in allele (*Pdgfra^PI3K^*) that renders PDGFRα unable to bind PI3K exhibit a cleft palate and die perinatally, among other defects (Klinghoffer et al., 2002; Fantauzzo and Soriano, 2014). This palatal clefting is less severe than the overt facial clefting phenotype found in *Pdgfra-*null and *Pdgfra^fl/fl^*;*Wnt1-Cre^+/Tg^* embryos (Soriano, 1997; Tallquist and Soriano, 2003; He and Soriano, 2013). While *Pdgfra^PI3K/PI3K^* embryos do not exhibit NCC migration defects at E9.5–E10.5 (He and Soriano, 2013), primary mouse embryonic palatal mesenchyme (MEPM) cells derived from E13.5 *Pdgfra^PI3K/PI3K^* embryos fail to proliferate in response to PDGF-AA ligand treatment (He and Soriano, 2013; Fantauzzo and Soriano, 2014). When the *Pdgfra^PI3K^* allele was combined with the *Pdgfrb^fl^* allele and the *Wnt1-Cre* driver, E13.5 double-homozygous mutant embryos had a complete facial clefting phenotype not observed in either single-homozygous mutant (Fantauzzo and Soriano, 2016). Further, addition of a single *Pdgfrb^fl^* allele worsened the midline defects found in *Pdgfra^PI3K/PI3K^* skeletons at E16.5; *Pdgfra^PI3K/PI3K^*;*Pdgfrb^+/fl^*;*Wnt1-Cre^+/Tg^* skeletons further exhibited nasal cartilage that was clefted and upturned, a widening of the space between the premaxilla bones and a broader skull (Fantauzzo and Soriano, 2016), similar to the frontonasal defects observed in *Pdgfra^fl/fl^*;*Wnt1-Cre^+/Tg^* embryos (Tallquist and Soriano, 2003; He and Soriano, 2013). Importantly, however, it could not be determined from this study whether the double-homozygous mutant phenotypes observed past mid-gestation were more severe than those exhibited by single-homozygous mutant embryos because of cell-autonomous or non-cell-autonomous effects on the NCC lineage.

To examine the effect of ablating both *Pdgfra* and *Pdgfrb* in the murine NCC lineage on earlier craniofacial development and to determine the cellular mechanisms by which the observed phenotypes arise, we analyzed an allelic series of mutant embryos. Our results confirm a genetic interaction between the two receptors in this lineage and demonstrate that PDGFRα plays a predominant role in cNCC migration whereas PDGFRβ exerts its effect primarily through the regulation of proliferation in the facial mesenchyme past mid-gestation.

## Materials and Methods

### Mouse Strains

All animal experimentation was approved by the Institutional Animal Care and Use Committee of the University of Colorado Anschutz Medical Campus. *Pdgfra^tm8Sor^* mice (Tallquist and Soriano, 2003), referred to in the text as *Pdgfra^fl^*; *Pdgfrb^tm11Sor^* mice (Schmahl et al., 2008), referred to in the text as *Pdgfrb^fl^*; *H2afv^Tg(Wnt1-cre)11Rth^* mice (Danielian et al., 1998), referred to in the text as *Wnt1-Cre^Tg^*; and *Gt(ROSA)26Sor^tm4(ACTB-tdTomato,-EGFP)Luo^* mice (Muzumdar et al., 2007), referred to in the text as *ROSA26^mTmG^*, were maintained on a 129S4 coisogenic genetic background. Statistical analyses of Mendelian inheritance were performed with the GraphPad QuickCalcs data analysis resource (GraphPad Software, Inc., La Jolla, CA, United States) using a *χ*^2^ test. Statistical analyses of litter sizes were performed with Prism 8 (GraphPad Software, Inc.) using a two-tailed, unpaired *t*-test with Welch’s correction.

### Morphological Analysis

Embryos were dissected at multiple timepoints (day of plug considered 0.5 days) in 1x phosphate buffered saline (PBS) and fixed overnight at 4°C in 4% paraformaldehyde (PFA) in PBS. Embryos were photographed using an Axiocam 105 color digital camera (Carl Zeiss, Inc., Thornwood, NY, United States) fitted onto a Stemi 508 stereo microscope (Carl Zeiss, Inc.). Distances between nasal pits and heights of heads were measured using Photoshop software v 21.1.1 (Adobe, San Jose, CA, United States). The normalized distance between nasal pits was calculated by dividing the distance between nasal pits by the height of the head from the anterior surface of the forebrain to the posterior surface of pharyngeal arch 1. Statistical analyses were performed on all embryos represented in graphs, regardless of somite pair number, with Prism 8 (GraphPad Software, Inc.) using a two-tailed, unpaired *t*-test with Welch’s correction and Welch and Brown-Forsythe ANOVA tests.

### Whole-Mount DAPI Staining

Whole-mount 4',6-diamidino-2-phenylindole (DAPI) staining was performed according to a previously published protocol (Sandell et al., 2012), except that staining was performed with 10 μg/ml DAPI (Sigma-Aldrich Corp., St. Louis, MO, United States) for 1 h at room temperature. Embryos were photographed using an Axiocam 506 mono digital camera (Carl Zeiss, Inc.) fitted onto an Axio Observer 7 fluorescence microscope (Carl Zeiss, Inc.). For lateral views of NCC streams, embryos were positioned on their sides at identical angles in glass-bottom dishes. Images were acquired and analyzed from both sides of each embryo using identical lamp intensities and exposure times. Extended depth of focus was applied to z-stacks using ZEN Blue software (Carl Zeiss, Inc.) to generate images with the maximum depth of field. Anterior-posterior heights and dorsal-ventral lengths of NCC streams in at least three embryos per genotype per timepoint were measured using ZEN Blue software (Carl Zeiss, Inc.). NCC stream height was measured at the tallest part of the middle of the stream. NCC stream length was measured from the dorsal-most edge of the NCC stream where cell condensation was apparent to the dorsal border of each pharyngeal arch, as determined by pharyngeal pouch morphology. The normalized distance of NCC streams was calculated by dividing the stream height or length by the height of the head from the crown to the posterior surface of pharyngeal arch 1. NCC stream bifurcations were assessed per stream and defined as errant holes and/or forking in the stream. NCC stream intermingling was assessed between streams entering pharyngeal arches 1–2 and between streams entering pharyngeal arches 3–4, and defined as two streams joining abnormally as compared to streams in control embryos. An Unsharp Mask was applied to select images of NCC streams at E10.5 using ImageJ software (version 2.0.0-rc-69/1.52p; National Institutes of Health) with radius 40 pixels and mask weight 0.90. For quantification of green fluorescent protein (GFP) intensity in the facial processes, embryos were positioned face-down at identical angles in 0.5% agarose-filled polystyrene dishes. Images were acquired using identical lamp intensities and exposure times. Extended depth of focus was applied to z-stacks using ZEN Blue software (Carl Zeiss, Inc.) to generate images with the maximum depth of field. GFP signal was measured in frontal views of at least three embryos per genotype per timepoint using ImageJ software (version 2.0.0-rc-69/1.52p; National Institutes of Health). For each embryo, the entire head was selected as the region of interest (ROI) to be measured. Values for integrated density for each ROI were recorded and normalized to the mean background value. For each embryo, the mean gray value for each of three separate regions surrounding but apart from the embryo were measured and averaged to obtain the mean background value. Relative fluorescence units were calculated using the following formula: corrected total fluorescence = integrated density – (ROI area × mean background). Statistical analyses were performed on all embryos represented in graphs, regardless of somite pair number, with Prism 8 (GraphPad Software, Inc.) using a two-tailed, unpaired *t*-test with Welch’s correction and Welch and Brown-Forsythe ANOVA tests.

### TUNEL Assay

Embryos were fixed in 4% PFA in PBS and infiltrated with 30% sucrose in PBS before being mounted in O.C.T. compound (Sakura Finetek United States Inc., Torrance, CA, United States). Sections (8 μm) were deposited on glass slides. Apoptotic cells were identified using the *In Situ* Cell Death Detection Kit, Fluorescein (Sigma-Aldrich Corp.) according to the manufacturer’s instructions for the treatment of cryopreserved tissue sections. Sections were mounted in VECTASHIELD® Antifade Mounting Medium with DAPI (Vector Laboratories, Burlingame, CA, United States) and photographed using an Axiocam 506 mono digital camera (Carl Zeiss, Inc.) fitted onto an Axio Observed 7 fluorescence microscope (Carl Zeiss, Inc.). All positive signals were confirmed by DAPI staining. The percentage of terminal deoxynucleotidyl transferase-mediated dUTP nick end labeling (TUNEL)-positive cells was determined in three embryos per genotype per timepoint, with up to four sections analyzed per individual embryo. Analyzed sections within a given embryo were 5–10 sections apart, representing a distance of 40–80 μm. Graphed data represent averages from three independent embryos per timepoint. Statistical analyses were performed on values from individual sections with Prism 8 (GraphPad Software, Inc.) using a two-tailed, unpaired *t*-test with Welch’s correction and Welch and Brown-Forsythe ANOVA tests.

### Ki67 Immunofluorescence Analysis

Sections (8 μm) of PFA-fixed, sucrose-infiltrated, O.C.T.-mounted embryos were deposited on glass slides. Sections were fixed in 4% PFA in PBS with 0.1% Triton X-100 for 10 min and washed in PBS with 0.1% Triton-X 100. Sections were blocked for 1 h in 5% normal donkey serum (Jackson ImmunoResearch Inc., West Grove, PA, United States) in PBS and incubated overnight at 4°C in anti-Ki67 primary antibody (1:300; Invitrogen, Carlsbad, CA, United States) in 1% normal donkey serum in PBS. After washing in PBS, sections were incubated in Alexa Fluor 488-conjugated donkey anti-rabbit secondary antibody (1:1,000; Invitrogen) diluted in 1% normal donkey serum in PBS with 2 μg/ml DAPI (Sigma-Aldrich Corp.) for 1 h. Sections were mounted in Aqua Poly/Mount mounting medium (Polysciences, Inc., Warrington, PA, United States) and photographed using an Axiocam 506 mono digital camera (Carl Zeiss, Inc.) fitted onto an Axio Observer 7 fluorescence microscope (Carl Zeiss, Inc.). All positive signals were confirmed by DAPI staining. The percentage of Ki67-positive cells was determined in three embryos per genotype per timepoint, with up to four sections analyzed per individual embryo. Analyzed sections within a given embryo were 5–10 sections apart, representing a distance of 40–80 μm. Graphed data represent averages from three independent embryos per timepoint. Statistical analyses were performed on values from individual sections with Prism 8 (GraphPad Software, Inc.) using a two-tailed, unpaired *t*-test with Welch’s correction and Welch and Brown-Forsythe ANOVA tests.

### Cell Culture and Growth Assays

Primary MEPM cells were isolated from the palatal shelves of embryos dissected at E13.5 in PBS and cultured in medium [Dulbecco’s modified Eagle’s medium (GIBCO, Invitrogen) supplemented with 50 U/ml penicillin (GIBCO), 50 μg/ml streptomycin (GIBCO), and 2 mM L-glutamine (GIBCO)] containing 10% fetal bovine serum (FBS; HyClone Laboratories, Inc., Logan, UT, United States) as previously described (Bush and Soriano, 2010). For cell growth assays, 11,500 passage 2 MEPM cells were seeded into wells of a 24-well plate and cultured in medium containing 10% FBS. After 24 h, medium was aspirated and replaced with fresh medium containing 10% FBS (growth medium) or 0.1% FBS (starvation medium).

After 1, 2, 3, 4, and 6 total days in culture, cells were subsequently fixed in 4% PFA in PBS, stained with 0.1% crystal violet in 10% ethanol, extracted with 10% acetic acid, and the absorbance measured at 590 nm. Data represent results from three independent trials, each consisting of MEPM cells derived from one heterozygous embryo and at least one conditional knock-out littermate. Statistical analyses were performed with Prism 8 (GraphPad Software, Inc.) using a two-tailed, unpaired *t*-test with Welch’s correction and Welch and Brown-Forsythe ANOVA tests.

## Results

### *Pdgfra* and *Pdgfrb* Genetically Interact in the NCC Lineage

To examine the effect of ablating both *Pdgfra* and *Pdgfrb* in the NCC lineage on mid-gestation craniofacial development, we intercrossed *Pdgfra^fl/fl^*;*Pdgfrb^fl/fl^* mice with *Pdgfra^+/fl^*;*Pdgfrb^+/fl^*;*Wnt1-Cre^+/Tg^* mice and harvested the resulting progeny at E10.5 for gross morphological examination. Double-homozygous mutant embryos were recovered at Mendelian frequencies at this timepoint (16 embryos vs. 14 expected embryos out of 109 total, *χ*^2^ two-tailed *p* = 0.4915; Table 1). A small percentage of embryos across several of the eight allele combinations from the intercrosses exhibited an abnormal head shape due to a misshapen forebrain and/or midbrain, blebbing of the surface ectoderm in the facial region and/or facial hemorrhaging (Table 1). Further, 18% of *Pdgfra^fl/fl^*;*Pdgfrb^+/fl^*;*Wnt1-Cre^+/Tg^* embryos displayed ventral body wall closure defects (*n* = 11; Table 1).

**Table 1:** Phenotypes of E10.5 embryos from intercrosses of *Pdgfra^fl/fl^*;*Pdgfrb^fl/fl^* mice with *Pdgfra^+/fl^*;*Pdgfrb^+/fl^*;*Wnt1-Cre^+/Tg^* mice.

Genotype	Expected	Observed	Normal	Dead	Abnormal head	Facial bleb	Facial hemorrhage	Body wall closure defects
*α^+/fl^;β^+/fl^;W1C^+/+^*	0.125	0.092	10/10	1	0/10	0/10	0/10	0/10
*α^+/fl^;β^+/fl^;W1C^+/Tg^*	0.125	0.192	19/21	2	0/21	2/21	0/21	0/21
*α^+/fl^;β^fl/fl^;W1C^+/+^*	0.125	0.117	13/14	0	1/14	0/14	0/14	0/14
*α^+/fl^;β^fl/fl^;W1C^+/Tg^*	0.125	0.075	7/9	0	1/9	0/9	1/9	0/9
*α^fl/fl^;β^+/fl^;W1C^+/+^*	0.125	0.142	14/14	3	0/14	0/14	0/14	0/14
*α^fl/fl^;β^+/fl^;W1C^+/Tg^*	0.125	0.117	8/11	3	1/11	1/11	1/11	2/11
*α^fl/fl^;β^fl/fl^;W1C^+/+^*	0.125	0.117	12/14	0	1/14	0/14	1/14	0/14
*α^fl/fl^;β^fl/fl^;W1C^+/Tg^*	0.125	0.150	13/16	2	1/16	1/16	1/16	0/16

**Figure 1:**
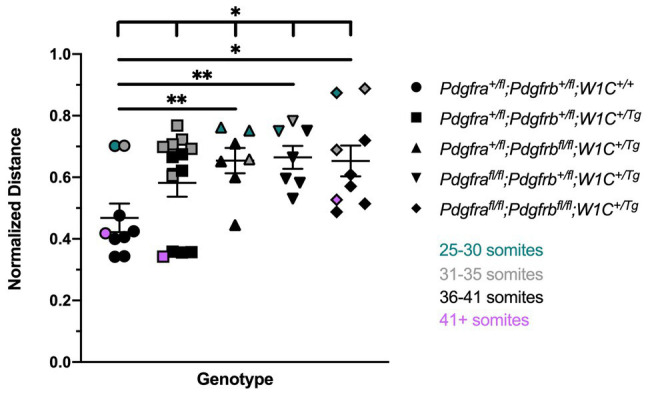
Ablation of *Pdgfra* and *Pdgfrb* in the neural crest cell (NCC) lineage leads to increased distances between the nasal pits at mid-gestation. Scatter dot plot depicting the normalized distance between nasal pits across five genotypes at E10.5. Data are presented as mean ± SEM. *p < 0.05; **p < 0.01. Colors correspond to number of somite pairs in assayed embryos.

We next measured the distance between nasal pits, normalized to the height of the head, in E10.5 embryos as a readout of defects at the facial midline, revealing a significant difference in measurements across one control (*Pdgfra^+/fl^*;*Pdgfrb^+/fl^*;*Wnt1-Cre^+/+^*) and the four experimental genotypes containing the *Wnt1-Cre* transgene (Welch’s ANOVA test *p* = 0.0390; Brown-Forsythe ANOVA test *p* = 0.0195). The distance between nasal pits was significantly increased in *Pdgfra^+/fl^*;*Pdgfrb^fl/fl^*;*Wnt1-Cre^+/Tg^* embryos (0.6542 ± 0.04112, *p* = 0.0095), *Pdgfra^fl/fl^*;*Pdgfrb^+/fl^*;*Wnt1-Cre^+/Tg^* embryos (0.6648 ± 0.03722, *p* = 0.0052), and double-homozygous mutant embryos (0.6532 ± 0.05014, *p* = 0.0155) compared to control *Pdgfra^+/fl^*;*Pdgfrb^+/fl^*;*Wnt1-Cre^+/+^* embryos (0.4682 ± 0.04628; Figure 1). While double-heterozygous mutant embryos had a larger distance between nasal pits than control embryos, this difference was not statistically significant (Figure 1). Interestingly, the greatest distance between nasal pits was observed in *Pdgfra^fl/fl^*;*Pdgfrb^+/fl^*;*Wnt1-Cre^+/Tg^* embryos, though this distance was not significantly different between these and double-homozygous mutant embryos (Figure 1).

**Figure 2:**
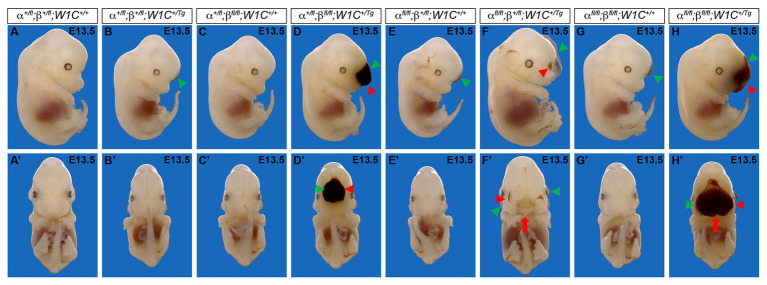
Ablation of *Pdgfra* and *Pdgfrb* in the NCC lineage results in facial clefting, blebbing, and hemorrhaging at E13.5. **(A–H')** Gross morphology of E13.5 embryos resulting from intercrosses of *Pdgfra^fl/fl^*;*Pdgfrb^fl/fl^* mice with *Pdgfra^+/fl^*;*Pdgfrb^+/fl^*;*Wnt1-Cre^+/Tg^* mice as viewed laterally **(A–H)** and frontally **(A'–H')**. *Pdgfra^fl/fl^*;*Pdgfrb^+/fl^*;*Wnt1-Cre^+/Tg^* and double-homozygous mutant embryos exhibited an overt facial cleft (red arrow). Facial blebbing (green arrowheads) and facial hemorrhaging (red arrowheads) were also detected among embryos possessing a variety of allele combinations.

To determine whether the above craniofacial phenotypes persisted or worsened at later timepoints, embryos were harvested at E13.5 from the same intercrosses (Figure 2). While the presence of the *Wnt1-Cre* transgene always exacerbated E13.5 facial phenotypes, facial blebbing was detected in a subset of embryos upon combination of at least three out of four conditional alleles in the absence of the *Wnt1-Cre* transgene, reaching a prevalence of 83% in *Pdgfra^fl/fl^*;*Pdgfrb^+/fl^*;*Wnt1-Cre^+/+^* embryos (*n* = 12; Table 2; Figures 2E,G). Further, facial hemorrhaging was noted in approximately 15% of *Pdgfra^fl/fl^*;*Pdgfrb^+/fl^*;*Wnt1-Cre^+/+^* embryos (*n* = 12) and double-homozygous floxed embryos without Cre (*n* = 14; Table 2). These results indicate that one or both of the conditional alleles are hypomorphic. However, the fact that only 8% of *Pdgfra^+/fl^*;*Pdgfrb^fl/fl^*;*Wnt1-Cre^+/+^* embryos exhibited facial blebbing (*n* = 13), and none of these embryos exhibited facial hemorrhaging (*n* = 13), combined with the finding that the prevalence of these phenotypes was comparable between *Pdgfra^fl/fl^*;*Pdgfrb^+/fl^*;*Wnt1-Cre^+/+^* embryos and double-homozygous floxed embryos without Cre, in which 64% (*n* = 14) and 14% (*n* = 14) of embryos exhibited facial blebbing and hemorrhaging, respectively (Table 2), indicates that the *Pdgfrb^fl^* allele is not hypomorphic. Double-homozygous mutant embryos were recovered at Mendelian frequencies at this timepoint as well (8 embryos vs. 12 expected embryos out of 93 total, *χ*^2^ two-tailed *p* = 0.2557; Table 2). A fully-penetrant, overt facial clefting phenotype was observed in *Pdgfra^fl/fl^*;*Pdgfrb^+/fl^*;*Wnt1-Cre^+/Tg^* embryos (100%; *n* = 12; Figure 2F') and double-homozygous mutant embryos (100%; *n* = 8; Figure 2H'), though not in any of the other six allele combinations from the intercrosses (*n* = 73; Table 2). Facial blebbing was detected in the majority of embryos among the four genotypes containing the *Wnt1-Cre* allele and was fully penetrant in *Pdgfra^fl/fl^*;*Pdgfrb^+/fl^*;*Wnt1-Cre^+/Tg^* embryos (100%; *n* = 12; Table 2; Figures 2B,D,D',F,F',H,H'). Similarly, facial hemorrhaging was observed in the majority of embryos containing at least three out of four conditional alleles in combination with the *Wnt1-Cre* transgene and was fully penetrant in double-homozygous mutant embryos (100%, *n* = 8; Table 2; Figures 2D,D',F,F',H,H'). Together, these results demonstrate that *Pdgfra* and *Pdgfrb* genetically interact in the NCC lineage, with PDGFRα playing a more predominant role in NCC-mediated craniofacial development.

**Table 2:** Phenotypes of E13.5 embryos from intercrosses of *Pdgfra^fl/fl^*;*Pdgfrb^fl/fl^* mice with *Pdgfra^+/fl^*;*Pdgfrb^+/fl^*;*Wnt1-Cre^+/Tg^* mice.

Genotype	Expected	Observed	Normal	Dead	Facial cleft	Facial bleb	Facial hemorrhage
*α^+/fl^;β^+/fl^;W1C^+/+^*	0.125	0.077	5/5	3	0/5	0/5	0/5
*α^+/fl^;β^+/fl^;W1C^+/Tg^*	0.125	0.173	5/16	2	0/16	10/16	2/16
*α^+/fl^;β^fl/fl^;W1C^+/+^*	0.125	0.135	12/13	1	0/13	1/13	0/13
*α^+/fl^;β^fl/fl^;W1C^+/Tg^*	0.125	0.154	2/13	3	0/13	11/13	9/13
*α^fl/fl^;β^+/fl^;W1C^+/+^*	0.125	0.125	2/12	1	0/12	10/12	2/12
*α^fl/fl^;β^+/fl^;W1C^+/Tg^*	0.125	0.115	0/12	0	12/12	12/12	9/12
*α^fl/fl^;β^fl/fl^;W1C^+/+^*	0.125	0.144	5/14	1	0/14	9/14	2/14
*α^fl/fl^;β^fl/fl^;W1C^+/Tg^*	0.125	0.077	0/8	0	8/8	7/8	8/8

### PDGFRα and, to a Lesser Extent, PDGFRβ Regulate cNCC Stream Size and Shape

**Figure 3:**
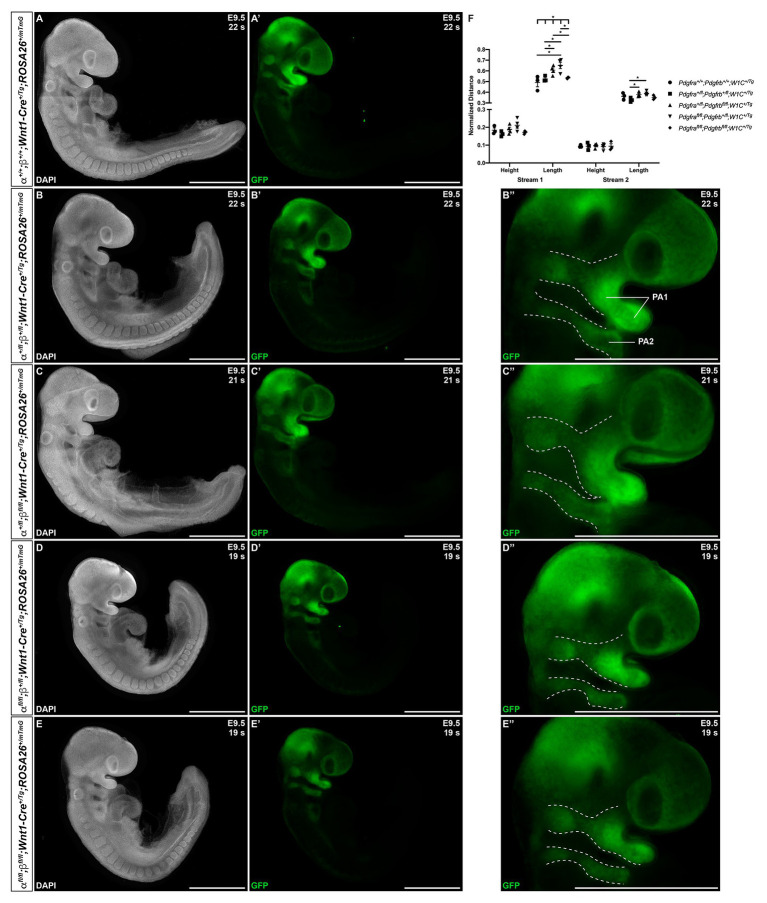
Ablation of *Pdgfra* and *Pdgfrb* in the NCC lineage leads to longer cranial NCC (cNCC) streams entering pharyngeal arch 1 (PA1) and pharyngeal arch 2 (PA2) at E9.5. **(A–E')** Lateral, whole-mount fluorescence images of 4’,6-diamidino-2-phenylindole (DAPI; **A–E**) and GFP **(A'–E')** expression across five genotypes at E9.5. **(B''–E'')** Zoomed-in images of GFP expression in cNCC streams (outlined by dotted lines) entering PA1 and PA2. PA1, pharyngeal arch 1; PA2, pharyngeal arch 2. **(F)** Scatter dot plot depicting the normalized anterior-posterior heights and dorsal-ventral lengths of cNCC streams entering PA1 and PA2 across five genotypes at E9.5. Data are presented as mean ± SEM. *p < 0.05.

We next introduced the *ROSA26^mTmG^* double-fluorescent Cre reporter allele (Muzumdar et al., 2007) into the above intercrosses to examine the timing, extent, and pattern of NCC migration at E9.5–E10.5. Whereas streams entering pharyngeal arches 1 (PA1) and 2 (PA2) were readily apparent in all embryos assayed at E9.5 (Figures 3A–E''), there was a trend for the stream entering PA1 to be taller along the anterior-posterior axis in *Pdgfra^+/fl^*;*Pdgfrb^fl/fl^*;*Wnt1-Cre^+/Tg^* embryos and especially *Pdgfra^fl/fl^*;*Pdgfrb^+/fl^*;*Wnt1-Cre^+/Tg^* embryos than in control *Pdgfra^+/+^*;*Pdgfrb^+/+^*;*Wnt1-Cre^+/Tg^* embryos (Figure 3F). The dorsal-ventral length of the stream entering PA1 was significantly different across one control and the four experimental genotypes (Brown-Forsythe ANOVA test *p* = 0.0137). Further, the length of this stream was significantly longer in *Pdgfra^+/fl^*;*Pdgfrb^fl/fl^*;*Wnt1-Cre^+/Tg^* embryos (0.6069 ± 0.02081) compared to double-heterozygous mutant embryos (0.5252 ± 0.01524, *p* = 0.0252) and double-homozygous mutant embryos (0.5352 ± 0.004245, *p* = 0.0383), and in *Pdgfra^fl/fl^*;*Pdgfrb^+/fl^*;*Wnt1-Cre^+/Tg^* embryos (0.6511 ± 0.03018) compared to control *Pdgfra^+/+^*;*Pdgfrb^+/+^*;*Wnt1-Cre^+/Tg^* embryos (0.4907 ± 0.03919, *p* = 0.0304), double-heterozygous mutant embryos (0.5252 ± 0.01524, *p* = 0.0179), and double-homozygous mutant embryos (0.5352 ± 0.004245, *p* = 0.0298; Figure 3F). While the anterior-posterior height of the stream entering PA2 was not significantly different across genotypes, the dorsal-ventral length of the stream entering PA2 was significantly longer in *Pdgfra^+/fl^*;*Pdgfrb^fl/fl^*;*Wnt1-Cre^+/Tg^* embryos (0.3789 ± 0.01033, *p* = 0.0269) and *Pdgfra^fl/fl^*;*Pdgfrb^+/fl^*;*Wnt1-Cre^+/Tg^* embryos (0.3886 ± 0.009503, *p* = 0.0144) compared to double-heterozygous mutant embryos (0.3285 ± 0.01171; Figure 3F). These results demonstrate that combined decreases in PDGFRα and PDGFRβ signaling lead to longer cNCC streams along the dorsal-ventral axis entering PA1 and PA2 at E9.5.

**Figure 4:**
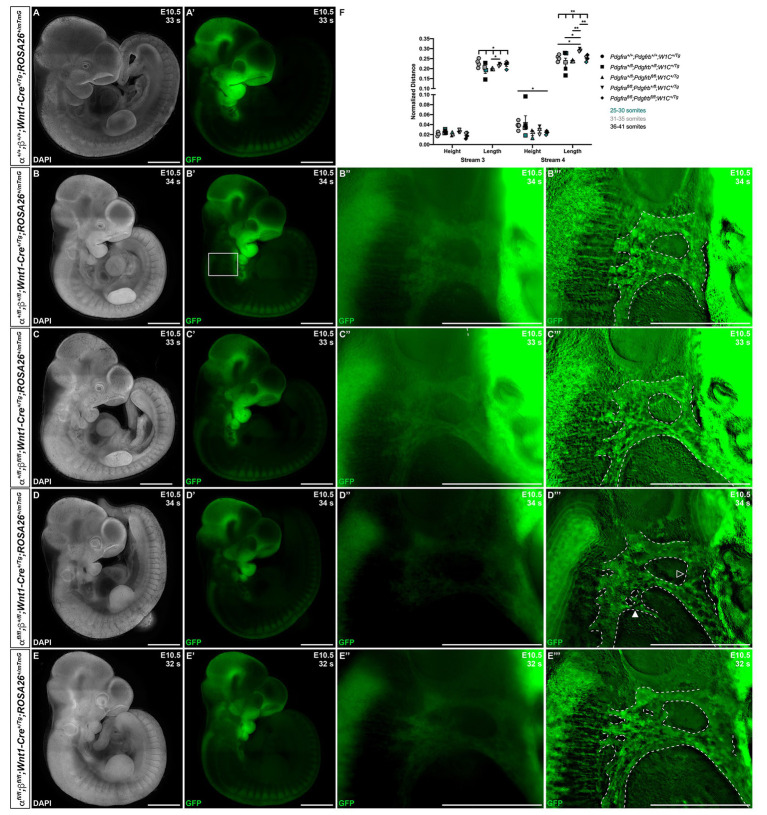
Ablation of *Pdgfra* and *Pdgfrb* in the NCC lineage results in longer cNCC streams entering PA3 and PA4 at E10.5, with increased incidences of stream bifurcations and intermingling. **(A–E')** Lateral, whole-mount fluorescence images of DAPI **(A–E)** and GFP **(A'–E')** expression across five genotypes at E10.5. **(B''–E''')** Zoomed-in images of GFP expression in cNCC streams (outlined by dotted lines) entering PA3 and PA4. Filled arrowhead indicates an example of a bifurcated cNCC stream. Unfilled arrowhead indicates an example of intermingling cNCC streams. **(F)** Scatter dot plot depicting the normalized anterior-posterior heights and dorsal-ventral lengths of cNCC streams entering PA3 and PA4 across five genotypes at E10.5. Data are presented as mean ± SEM. *p < 0.05; **p < 0.01. Colors correspond to number of somite pairs in assayed embryos.

At E10.5, whereas double-heterozygous mutant embryos (Figures 4B–B''') and *Pdgfra^+/fl^*;*Pdgfrb^fl/fl^*;*Wnt1-Cre^+/Tg^* embryos (Figures 4C–C''') appeared similar to control *Pdgfra^+/+^*;*Pdgfrb^+/+^*;*Wnt1-Cre^+/Tg^* embryos (Figures 4A,A') with clearly delineated NCC streams entering pharyngeal arches 3 (PA3) and 4 (PA4), *Pdgfra^fl/fl^*;*Pdgfrb^+/fl^*;*Wnt1-Cre^+/Tg^* embryos had streams with reduced GFP intensity and noticeable bifurcations (Figures 4D–D'''). Interestingly, the double-homozygous embryo phenotype was again less severe than that of *Pdgfra^fl/fl^*;*Pdgfrb^+/fl^*;*Wnt1-Cre^+/Tg^* embryos. Double-homozygous mutant embryos exhibited streams with intermediate GFP intensity and only mild bifurcations (Figures 4E–E'''). While the anterior-posterior height of the stream entering PA3 did not vary significantly across genotypes, the dorsal-ventral length of this stream was significantly different across one control and the four experimental genotypes (Welch’s ANOVA test *p* = 0.0403; Figure 4F). Further, the length of the stream entering PA3 was longer in *Pdgfra^fl/fl^*;*Pdgfrb^+/fl^*;*Wnt1-Cre^+/Tg^* embryos (0.2181 ± 0.003919) compared to *Pdgfra^+/fl^*;*Pdgfrb^fl/fl^*;*Wnt1-Cre^+/Tg^* embryos (0.1990 ± 0.002382, *p* = 0.0209; Figure 4F). The height of the stream entering PA4 was significantly shorter in double-homozygous mutant embryos (0.02393 ± 0.001587) compared to control *Pdgfra^+/+^*;*Pdgfrb^+/+^*;*Wnt1-Cre^+/Tg^* embryos (0.03852 ± 0.004417, *p* = 0.0390; Figure 4F). Finally, the length of the stream entering PA4 was significantly different across the five genotypes (Welch’s ANOVA test *p* = 0.0012), with *Pdgfra^fl/fl^*;*Pdgfrb^+/fl^*;*Wnt1-Cre^+/Tg^* embryos exhibiting significantly longer streams than those observed in every other genotype (Figure 4F).

**Figure 5:**
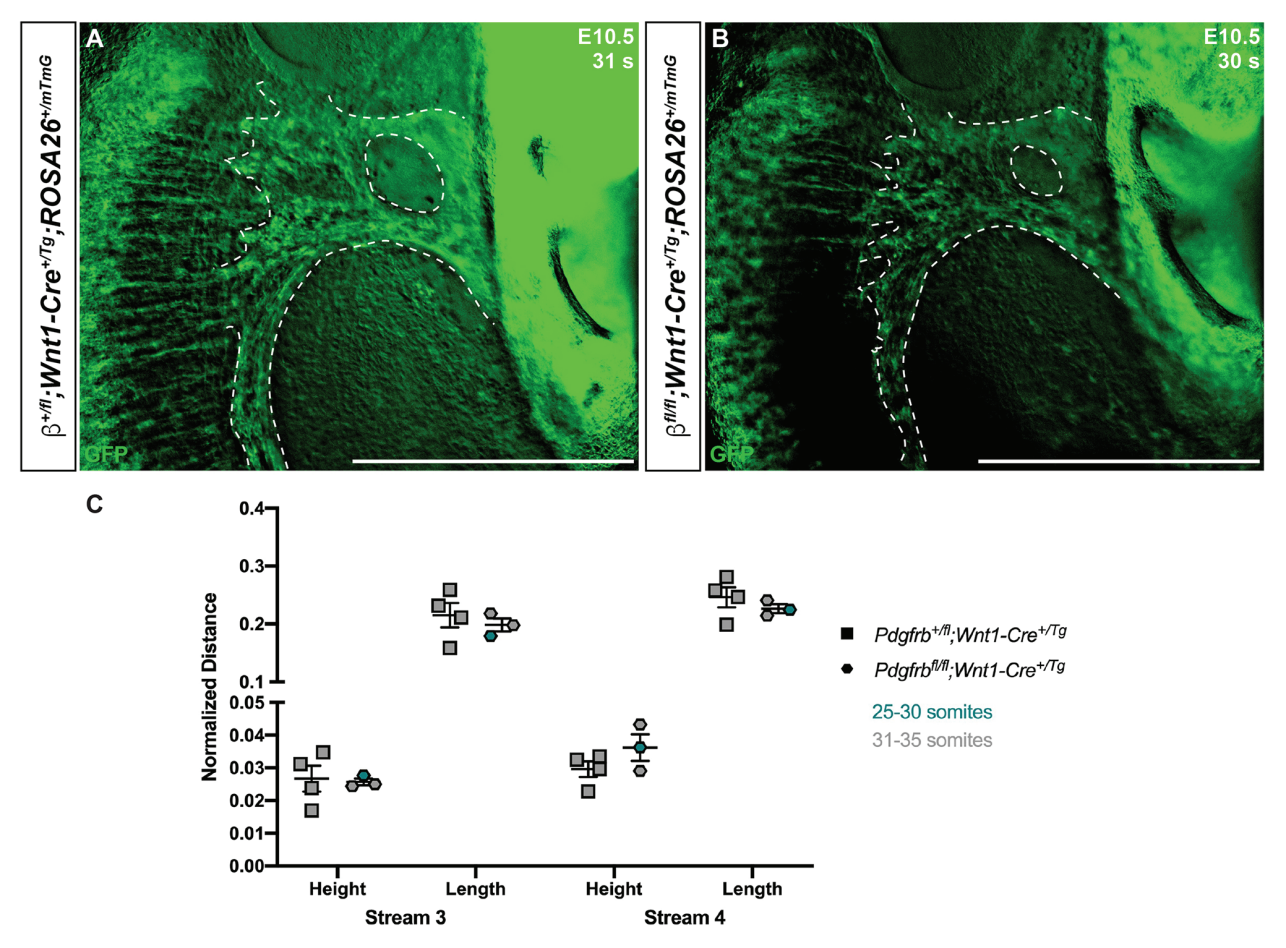
Ablation of *Pdgfrb* in the NCC lineage does not affect cNCC streams entering PA3 and PA4 at E10.5. **(A,B)** Zoomed-in images of GFP expression in cNCC streams (outlined by dotted lines) entering PA3 and PA4. **(C)** Scatter dot plot depicting the normalized anterior-posterior heights and dorsal-ventral lengths of cNCC streams entering PA3 and PA4 across two genotypes at E10.5. Data are presented as mean ± SEM. Colors correspond to number of somite pairs in assayed embryos.

Though we previously reported that *Pdgfrb^fl/fl^*;*Wnt1-Cre^+/Tg^* embryos do not have obvious defects in NCC migration into the facial processes and pharyngeal arches (Fantauzzo and Soriano, 2016), we conducted a more detailed analysis here, analyzing the anterior-posterior heights and dorsal-ventral lengths of the NCC streams entering PA3 and PA4 of *Pdgfrb^+/fl^*;*Wnt1-Cre^+/Tg^* and *Pdgfrb^fl/fl^*;*Wnt1-Cre^+/Tg^* embryos at E10.5, when we observed the most significant changes in NCC stream size in the above allelic series of embryos. These analyses revealed no significant differences in the size or shape of these streams between control and single conditional knock-out embryos (Figure 5).

The above allelic series of E10.5 embryos was then scored for bifurcations in streams entering PA3 and PA4 and intermingling of the two streams. For a handful of embryos with a relatively high number of somite pairs (≥39), the stream entering PA3 was no longer visible and hence was not assayed for bifurcation or intermingling with the stream entering PA4. The stream entering PA3 was not bifurcated in any of the double-heterozygous mutant embryos (*n* = 4), but was found to be bifurcated in 33% of *Pdgfra^+/fl^*;*Pdgfrb^fl/fl^*;*Wnt1-Cre^+/Tg^* embryos (*n* = 3), 50% of *Pdgfra^fl/fl^*;*Pdgfrb^+/fl^*;*Wnt1-Cre^+/Tg^* embryos (*n* = 2), and 67% of double-homozygous mutant embryos (*n* = 3; Table 3). Bifurcation of the stream entering PA4 was observed in 40% of double-heterozygous mutant embryos (*n* = 5), 67% of *Pdgfra^fl/fl^*;*Pdgfrb^+/fl^*;*Wnt1-Cre^+/Tg^* embryos (*n* = 3) and was fully penetrant in *Pdgfra^+/fl^*;*Pdgfrb^fl/fl^*;*Wnt1-Cre^+/Tg^* embryos (100%; *n* = 3) and double-homozygous mutant embryos (100%; *n* = 4; Table 3). Finally, the streams entering PA3 and PA4 were intermingled in 75% of double-heterozygous mutant embryos (*n* = 4) and in all *Pdgfra^+/fl^*;*Pdgfrb^fl/fl^*;*Wnt1-Cre^+/Tg^* embryos (100%; *n* = 3), *Pdgfra^fl/fl^*;*Pdgfrb^+/fl^*;*Wnt1-Cre^+/Tg^* embryos (100%; *n* = 2), and double-homozygous mutant embryos (100%; *n* = 3; Table 3). Taken together, the results at E10.5 indicate that combined decreases in PDGFRα and PDGFRβ signaling lead to longer cNCC streams with reduced GFP intensity along the dorsal-ventral axis entering PA3 and PA4, with increased incidences of stream bifurcations and intermingling.

**Table 3:** Bifurcation and intermingling of NCC streams entering PA3 and PA4 at E10.5.

Genotype	Bifurcated stream 3	Bifurcated stream 4	Intermingling of streams 3 and 4
*α^+/fl^;β^+/fl^;W1C^+/Tg^*	0/4	2/5	3/4
*α^+/fl^;β^fl/fl^;W1C^+/Tg^*	1/3	3/3	3/3
*α^fl/fl^;β^+/fl^;W1C^+/Tg^*	1/2	2/3	2/2
*α^fl/fl^;β^fl/fl^;W1C^+/Tg^*	2/3	4/4	3/3

**Figure 6:**
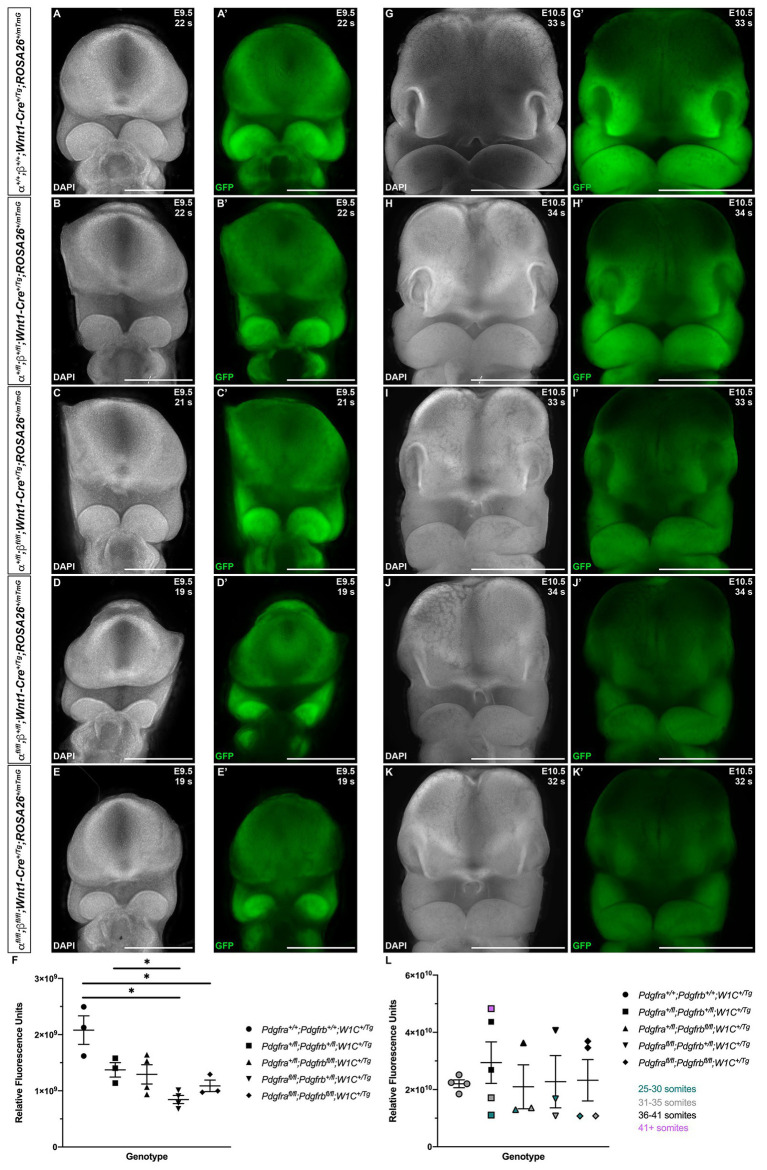
Ablation of *Pdgfra* and *Pdgfrb* in the NCC lineage leads to decreased NCC derivatives in the facial prominences at mid-gestation. **(A–K')** Frontal, whole-mount fluorescence images of DAPI **(A–E,G–K)** and GFP **(A'–E',G'–K')** expression across five genotypes at E9.5 **(A–E')** and E10.5 **(G–K')**. **(F)** Scatter dot plot depicting GFP fluorescence intensity across five genotypes at E9.5. Data are presented as mean ± SEM. *p < 0.05. **(L)** Scatter dot plot depicting GFP fluorescence intensity across five genotypes at E10.5. Data are presented as mean ± SEM. Colors correspond to number of somite pairs in assayed embryos.

Finally, to assess the extent of NCCs and their derivatives in the facial processes at E9.5 and E10.5, we quantified GFP expression in frontal views of the head in control *Pdgfra^+/+^*;*Pdgfrb^+/+^*;*Wnt1-Cre^+/Tg^* embryos and among embryos with the four experimental genotypes (Figure 6). At E9.5, there were noticeable decreases in GFP intensity in the facial processes of experimental embryos (Figures 6B'–E') compared to control embryos (Figure 6A'), particularly in the frontonasal and MxPs. GFP fluorescence values were significantly decreased in *Pdgfra^fl/fl^*;*Pdgfrb^+/fl^*;*Wnt1-Cre^+/Tg^* embryos (8.449 × 10^8^ ± 7.256 × 10^7^) compared to control *Pdgfra^+/+^*;*Pdgfrb^+/+^*;*Wnt1-Cre^+/Tg^* embryos (2.079 × 10^9^ ± 2.539 × 10^8^, *p* = 0.0317) and double-heterozygous mutant embryos (1.373 × 10^9^ ± 1.283 × 10^8^, *p* = 0.0325; Figure 6F). Moreover, while double-homozygous mutant embryos had higher GFP fluorescence values than *Pdgfra^fl/fl^*;*Pdgfrb^+/fl^*;*Wnt1-Cre^+/Tg^* embryos, GFP fluorescence was significantly decreased in double-homozygous mutant embryos (1.088 × 10^9^ ± 1.022 × 10^8^) compared to control *Pdgfra^+/+^*;*Pdgfrb^+/+^*;*Wnt1-Cre^+/Tg^* embryos (2.079 × 10^9^ ± 2.539 × 10^8^, *p* = 0.0448; Figure 6F). At E10.5, there was a marked decrease in GFP intensity in the facial processes of double-heterozygous mutant embryos (Figure 6H') compared to control *Pdgfra^+/+^*;*Pdgfrb^+/+^*;*Wnt1-Cre^+/Tg^* embryos (Figure 6G') and a further decrease in *Pdgfra^+/fl^*;*Pdgfrb^fl/fl^*;*Wnt1-Cre^+/Tg^* embryos (Figure 6I'), *Pdgfra^fl/fl^*;*Pdgfrb^+/fl^*;*Wnt1-Cre^+/Tg^* embryos (Figure 6J'), and double-homozygous mutant embryos (Figure 6K'). Not surprisingly, GFP fluorescence values increased with the number of somite pairs, as NCC progenitors proliferate and differentiate over time (Figure 6L). However, for embryos with 31–35 somite pairs, relative fluorescence units decreased as additional alleles were ablated, with *Pdgfra^fl/fl^*;*Pdgfrb^+/fl^*;*Wnt1-Cre^+/Tg^* and double-homozygous mutant embryos having the lowest, and essentially equal, GFP fluorescence values (Figure 6L). Collectively, our assessment of early facial phenotypes in the context of *Pdgfra* and *Pdgfrb* ablation demonstrates that signaling through these receptors contributes to several aspects of NCC activity, including stream size, stream shape and, ultimately, the extent of their derivatives in the facial prominences. Importantly, PDGFRα signaling appears to play a more predominant role in cNCC migration than PDGFRβ.

### PDGFRβ Plays a More Dominant Role in Proliferation of the Facial Mesenchyme Than PDGFRα Past Mid-Gestation

**Figure 7:**
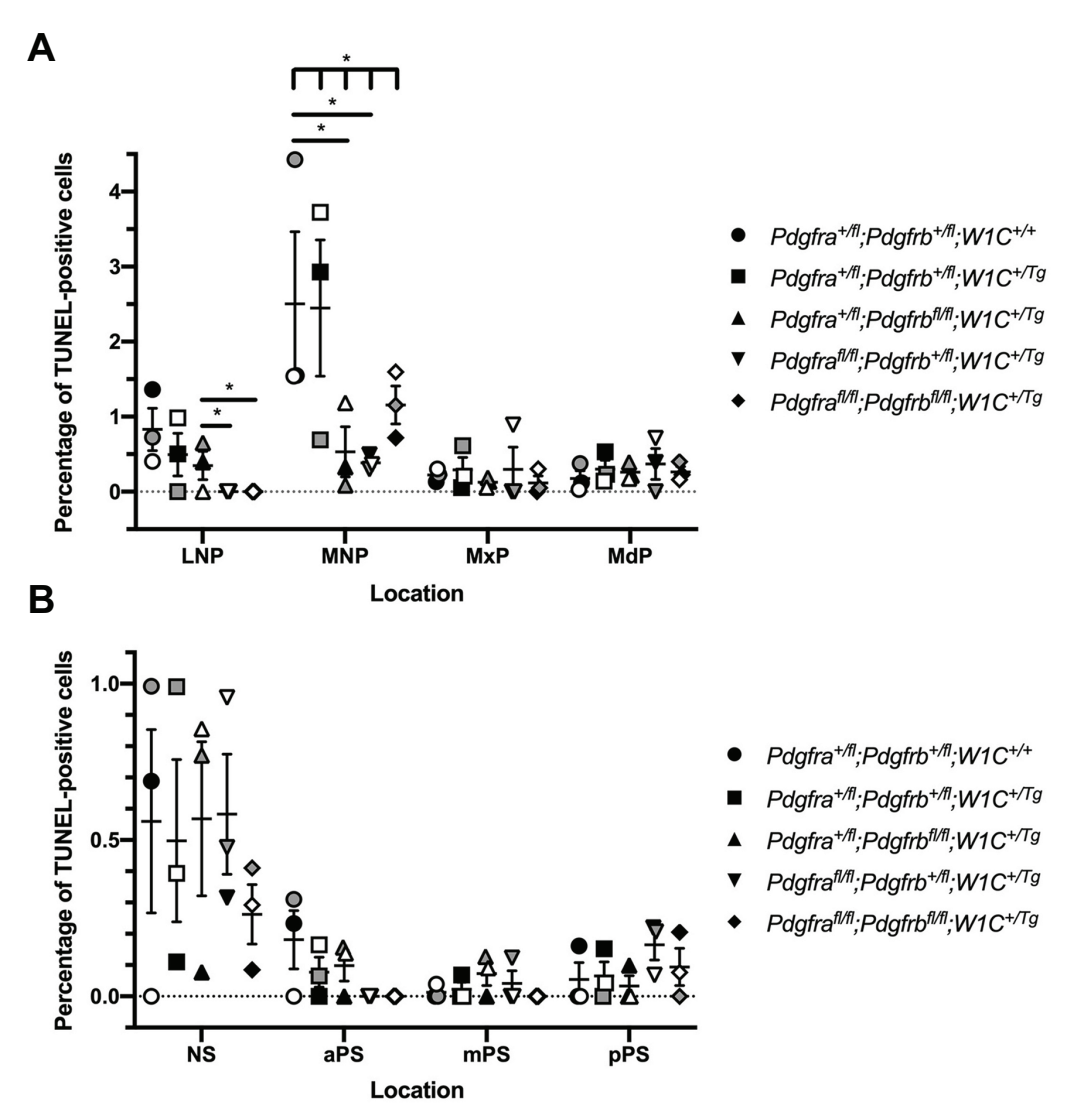
Neither PDGFRα nor PDGFRβ signaling plays a critical role in cNCC-derived facial mesenchyme survival during mid-gestation. **(A)** Scatter dot plot depicting the average percentage of terminal deoxynucleotidyl transferase-mediated dUTP nick end labeling (TUNEL)-positive cells per embryo in the nasal processes and facial prominences across five genotypes at E10.5. Data are presented as mean ± SEM. *p < 0.05. Shades correspond to independent experiments across three biological replicates. LNP, lateral nasal process; MNP, medial nasal process; MxP, maxillary prominence; and MdP, mandibular prominence. **(B)** Scatter dot plot depicting the average percentage of TUNEL-positive cells per embryo in the nasal septum (NS) and secondary palatal shelves across five genotypes at E13.5. Data are presented as mean ± SEM. Shades correspond to independent experiments across three biological replicates. NS, nasal septum; aPS, anterior secondary palatal shelves; mPS, middle secondary palatal shelves; and pPS, posterior secondary palatal shelves.

We next examined levels of cell death among one control, *Pdgfra^+/fl^*;*Pdgfrb^+/fl^*;*Wnt1-Cre^+/+^*, and the four experimental genotypes containing the *Wnt1-Cre* transgene *via* TUNEL. At E10.5, the percentage of TUNEL-positive cells was determined within the mesenchyme of the lateral and medial nasal processes, as well as the MxPs and MdPs. The percentage of TUNEL-positive cells was higher in the MNPs than the other locations at this timepoint for all genotypes (Figure 7A). Interestingly, in contrast to the other genotypes, no TUNEL-positive cells were found across any of the sections analyzed for *Pdgfra^fl/fl^*;*Pdgfrb^+/fl^*;*Wnt1-Cre^+/Tg^* embryos and double-homozygous mutant embryos in the lateral nasal processes (LNPs; Figure 7A). In the MNPs, both *Pdgfra^+/fl^*;*Pdgfrb^fl/fl^*;*Wnt1-Cre^+/Tg^* embryos (0.5328 ± 0.2316, *p* = 0.0212) and *Pdgfra^fl/fl^*;*Pdgfrb^+/fl^*;*Wnt1-Cre^+/Tg^* embryos (0.3792 ± 0.09148, *p* = 0.0137) had a significant decrease in apoptosis compared to control embryos (2.263 ± 0.5778). Further, there was a significant difference in the percentage of TUNEL-positive cells across all five genotypes at this location as assessed by a Welch’s ANOVA test (*p* = 0.0453; Figure 7A). While the level of cell death did not vary significantly between the five genotypes within the MxPs and MdPs, *Pdgfra^fl/fl^*;*Pdgfrb^+/fl^*;*Wnt1-Cre^+/Tg^* embryos had the highest percentages of TUNEL-positive cells at these locations (Figure 7A). In the MdP, there was a trend for each of the experimental genotypes to have a higher percentage of TUNEL-positive cells when compared to control embryos (Figure 7A). At E13.5, the percentage of TUNEL-positive cells was determined within the mesenchyme of the NS and anterior (aPS), middle (mPS), and posterior secondary palatal shelves (pPS). The percentage of TUNEL-positive cells was higher in the NS than in the secondary palatal shelves for all genotypes, consistent with the relatively high level of TUNEL-positive cells in the MNPs 3 days earlier at E10.5. Two genotypes, *Pdgfra^+/fl^*;*Pdgfrb^fl/fl^*;*Wnt1-Cre^+/Tg^* embryos and *Pdgfra^fl/fl^*;*Pdgfrb^+/fl^*;*Wnt1-Cre^+/Tg^* embryos, had a non-statistically significant increase in the percentage of TUNEL-positive cells compared to the control genotype at this location (Figure 7B). While the level of cell death did not vary significantly between the five genotypes within the secondary palatal shelves, there was a trend for each of the experimental genotypes to have a lower percentage of TUNEL-positive cells in the aPS when compared to control embryos (Figure 7B). In the mPS, three genotypes, double-heterozygous mutant embryos, *Pdgfra^+/fl^*;*Pdgfrb^fl/fl^*;*Wnt1-Cre^+/Tg^* embryos, and *Pdgfra^fl/fl^*;*Pdgfrb^+/fl^*;*Wnt1-Cre^+/Tg^* embryos, had a non-statistically significant increase in the percentage of TUNEL-positive cells compared to the control genotype (Figure 7B). Similarly, in the pPS, three genotypes, double-heterozygous mutant embryos, *Pdgfra^fl/fl^*;*Pdgfrb^+/fl^*;*Wnt1-Cre^+/Tg^* embryos, and double-homozygous mutant embryos, had a non-statistically significant increase in the percentage of TUNEL-positive cells compared to the control genotype (Figure 7B). Taken together, the combined TUNEL assay results demonstrate that neither PDGFRα nor PDGFRβ signaling plays a critical role in cNCC-derived facial mesenchyme survival during mid-gestation.

**Figure 8:**
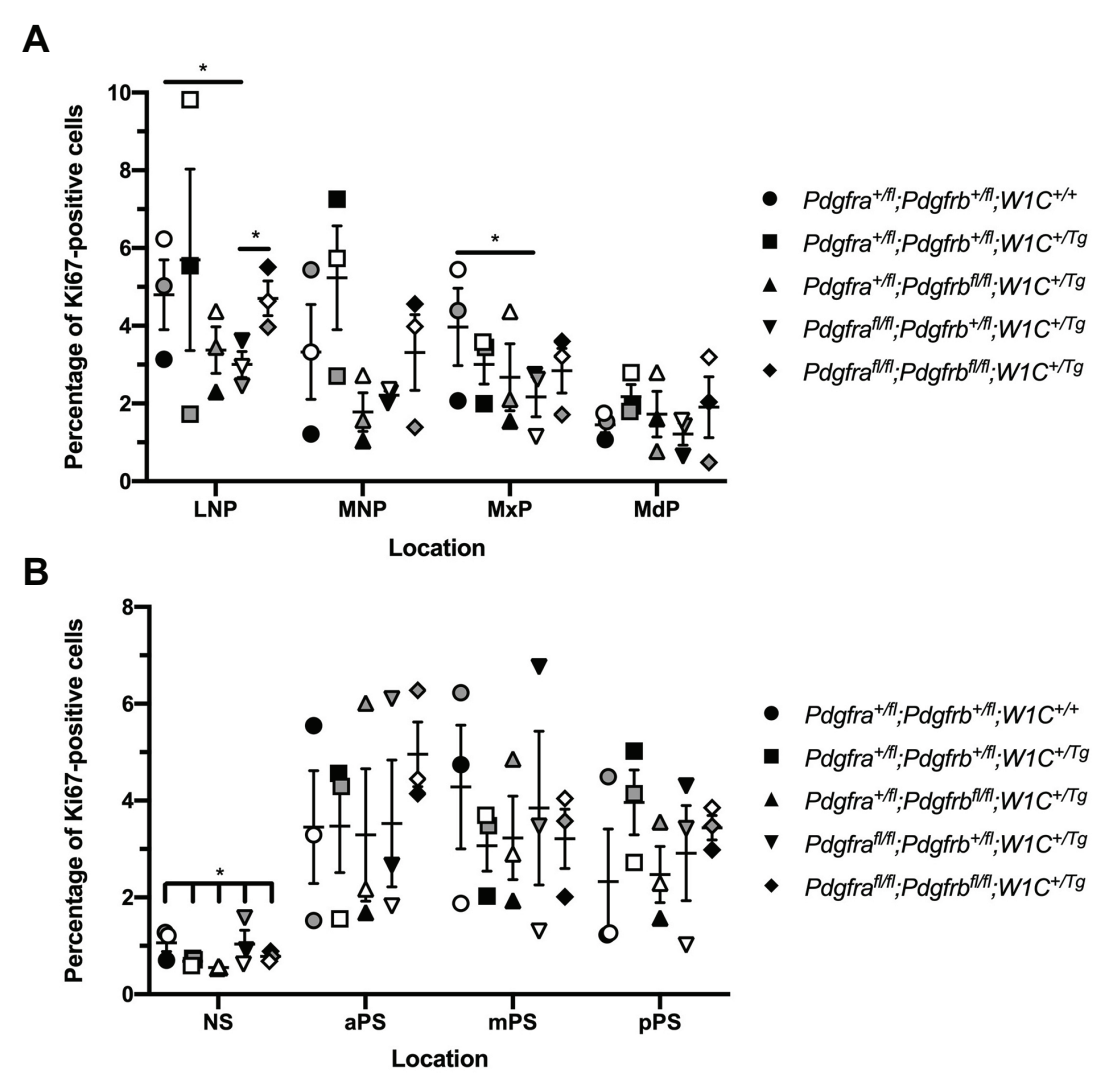
PDGFRα plays a more dominant role in proliferation of the craniofacial mesenchyme at E10.5, while PDGFRβ has a more pronounced effect at E13.5. **(A)** Scatter dot plot depicting the average percentage of Ki67-positive cells per embryo in the nasal processes and facial prominences across five genotypes at E10.5. Data are presented as mean ± SEM. *p < 0.05. Shades correspond to independent experiments across three biological replicates. LNP, lateral nasal process; MNP, medial nasal process; MxP, maxillary prominence; and MdP, mandibular prominence. **(B)** Scatter dot plot depicting the average percentage of Ki67-positive cells per embryo in the NS and secondary palatal shelves across five genotypes at E13.5. Data are presented as mean ± SEM. *p < 0.05. Shades correspond to independent experiments across three biological replicates. NS, nasal septum; aPS, anterior secondary palatal shelves; mPS, middle secondary palatal shelves; and pPS, posterior secondary palatal shelves.

We similarly examined levels of cell proliferation among one control, *Pdgfra^+/fl^*;*Pdgfrb^+/fl^*;*Wnt1-Cre^+/+^*, and the four experimental genotypes containing the *Wnt1-Cre* transgene *via* Ki67 immunofluorescence analysis. At E10.5, the percentage of Ki67-positive cells was determined within the mesenchyme of the lateral and medial nasal processes, as well as the MxPs and MdPs. The percentage of Ki67-positive cells was highest in the LNPs and lowest in the MdPs for all genotypes (Figure 8A). *Pdgfra^fl/fl^*;*Pdgfrb^+/fl^*;*Wnt1-Cre^+/Tg^* embryos exhibited a significant decrease in cell proliferation in the LNPs (2.882 ± 0.5367) compared to control embryos (5.003 ± 0.7518, *p* = 0.0450) and double-homozygous mutant embryos (4.687 ± 0.4514, *p* = 0.0368), as well as a significant decrease in the MxPs (2.168 ± 0.5133) compared to control embryos (4.350 ± 0.7249, *p* = 0.0494; Figure 8A). Interestingly, the percentage of Ki67-positive cells was consistently lower in *Pdgfra^+/fl^*;*Pdgfrb^fl/fl^*;*Wnt1-Cre^+/Tg^* embryos and *Pdgfra^fl/fl^*;*Pdgfrb^+/fl^*;*Wnt1-Cre^+/Tg^* embryos than double-homozygous mutant embryos at all locations at this timepoint (Figure 8A). As above with the TUNEL analysis, at E13.5, the percentage of Ki67-positive cells was determined within the mesenchyme of the NS and aPS, mPS, and pPS. The percentage of Ki67-positive cells was consistently lower in the NS than the secondary palatal shelves (Figure 8B). Though there were no significant differences in cell proliferation in pair-wise comparisons between genotypes in the NS, there was a significant difference in the percentage of Ki67-positive cells across all five genotypes as assessed by a Welch’s ANOVA test (*p* = 0.0453; Figure 8B). While the level of proliferation did not vary significantly between the five genotypes in the NS and along the anterior-posterior axis of the secondary palatal shelves, there were trends for each of the experimental genotypes to have a lower percentage of Ki67-positive cells in the NS and mPS when compared to these same locations in control embryos (Figure 8B). Intriguingly, *Pdgfra^+/fl^*;*Pdgfrb^fl/fl^*;*Wnt1-Cre^+/Tg^* embryos had a consistently lower percentage of Ki67-positive cells in the NS and aPS and mPS than *Pdgfra^fl/fl^*;*Pdgfrb^+/fl^*;*Wnt1-Cre^+/Tg^* embryos (Figure 8B). These findings indicate that both PDGFRα and PDGFRβ promote cell proliferation in the craniofacial mesenchyme, with PDGFRα playing a more predominant role in E10.5 facial structures and PDGFRβ potentially having a more pronounced effect on cell proliferation at E13.5.

**Figure 9:**
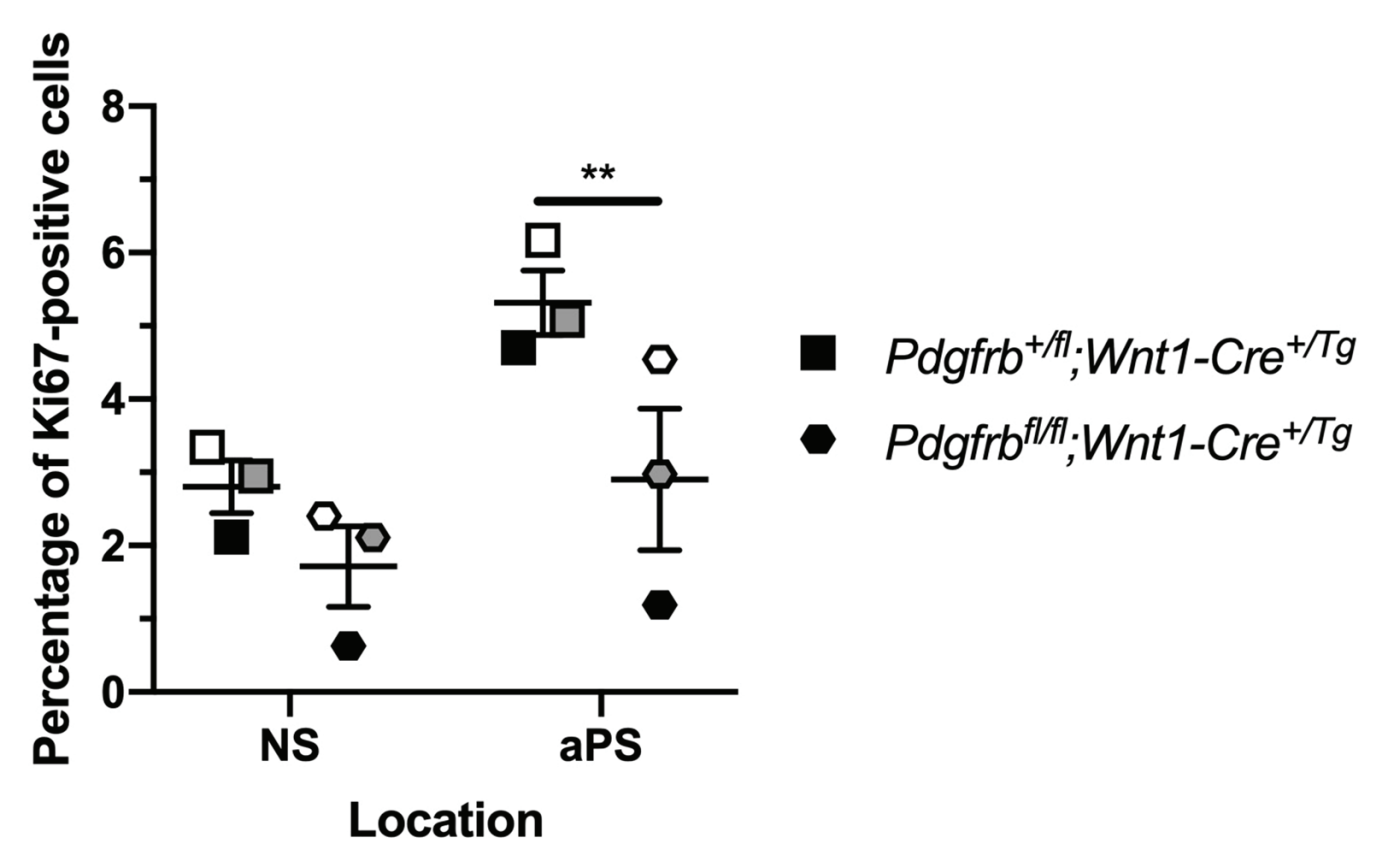
Ablation of *Pdgfrb* in the NCC lineage leads to decreased proliferation in the anterior secondary palatal shelves (aPS) at E13.5. Scatter dot plot depicting the average percentage of Ki67-positive cells per embryo in the NS and aPS in *Pdgfrb^+/fl^*;*Wnt1-Cre^+/Tg^* vs. *Pdgfrb^fl/fl^*;*Wnt1-Cre^+/Tg^* embryos at E13.5. Data are presented as mean ± SEM. **p < 0.01. Shades correspond to independent experiments across three biological replicates. NS, nasal septum; aPS, anterior secondary palatal shelves.

To confirm a role for PDGFRβ in promoting cell proliferation past mid-gestation, we determined the percentage of Ki67-positive cells within the mesenchyme of the NS and aPS of E13.5 *Pdgfrb^+/fl^*;*Wnt1-Cre^+/Tg^* vs. *Pdgfrb^fl/fl^*;*Wnt1-Cre^+/Tg^* embryos. The single conditional knock-out embryos exhibited a trend for decreased proliferation in the NS and a significant decrease in the percentage of Ki67-positive cells in the aPS (3.404 ± 0.5503) compared to heterozygous embryos (5.393 ± 0.3762, *p* = 0.0092; Figure 9).

**Figure 10:**
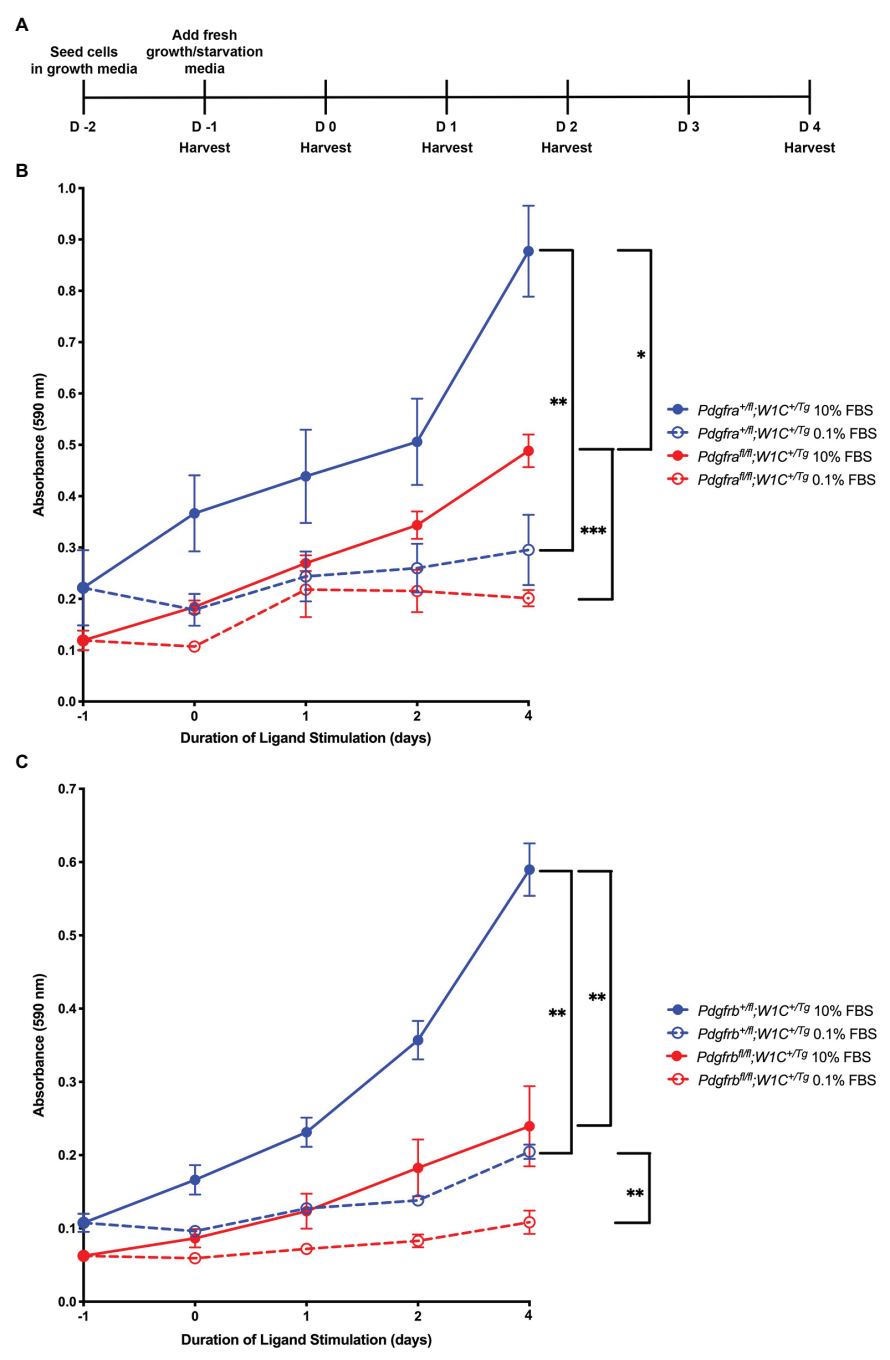
PDGFRβ plays a more dominant role in proliferation of primary MEPM cells than PDGFRα. **(A)** Experimental design for cell growth assays. **(B)** Line graph depicting absorbance values at 590 nm in *Pdgfra^+/fl^*;*Wnt1-Cre^+/Tg^* vs. *Pdgfra^fl/fl^*;*Wnt1-Cre^+/Tg^* primary MEPM cells across conditions. Data are presented as mean ± SEM. *p < 0.05; **p < 0.01; ***p < 0.001. **(C)** Line graph depicting absorbance values at 590 nm in *Pdgfrb^+/fl^*;*Wnt1-Cre^+/Tg^* vs. *Pdgfrb^fl/fl^*;*Wnt1-Cre^+/Tg^* primary MEPM cells across conditions. Data are presented as mean ± SEM. **p < 0.01.

We subsequently sought to determine the individual contribution of PDGFRα and PDGFRβ to proliferation of the craniofacial mesenchyme and to distinguish any potential proliferation defects from more wide-spread phenotypes observed upon ablation of *Pdgfra* or *Pdgfrb* in the NCC lineage. To do this, primary MEPM cells were dissected from E13.5 control (*Pdgfra^+/fl^*;*Wnt1-Cre^+/Tg^* or *Pdgfrb^+/fl^*;*Wnt1-Cre^+/Tg^*) and conditional knock-out (*Pdgfra^fl/fl^;Wnt1-Cre^+/Tg^* or *Pdgfrb^fl/fl^*;*Wnt1-Cre^+/Tg^*) littermate embryos for use in cell growth assays (Figure 10A). Primary MEPM cells are a faithful surrogate for embryonic facial mesenchyme, as wild-type cells express both PDGFRα and PDGFRβ as well as numerous additional *in vivo* palatal mesenchyme cell markers, and respond to stimulation with PDGF-AA, PDGF-BB, and PDGF-DD ligands (He and Soriano, 2013; Fantauzzo and Soriano, 2014, 2016, 2017; Vasudevan and Soriano, 2014; Vasudevan et al., 2015). Even after a single day in growth medium containing 10% FBS, control *Pdgfrb^+/fl^*;*Wnt1-Cre^+/Tg^* cells [0.1077 ± 0.01233 arbitrary units (AUs); Figure 10C] had grown about half as much as control *Pdgfra^+/fl^*;*Wnt1-Cre^+/Tg^* cells (0.2217 ± 0.07322 AU; Figure 10B). All cells grown in starvation medium containing 0.1% FBS, both control and conditional knock-out, immediately proliferated less than cells of the same genotypes grown in growth medium (Figures 10B,C). These trends continued after 5 days in culture, at which point significant differences were detected in all comparisons with the exception of *Pdgfrb^fl/fl^*;*Wnt1-Cre^+/Tg^* cells, which did not proliferate significantly more in medium containing 10% FBS than in medium containing 0.1% FBS (Figures 10B,C). Importantly, conditional knock-out cells consistently fared worse than their control counterparts in both growth and starvation medium, though this difference was more pronounced in *Pdgfrb^+/fl^*;*Wnt1-Cre^+/Tg^* vs. *Pdgfrb^fl/fl^*;*Wnt1-Cre^+/Tg^* cells following 6 days in culture. At this time, control *Pdgfra^+/fl^*;*Wnt1-Cre^+/Tg^* cells cultured in growth medium (0.8773 ± 0.08867 AU) had proliferated approximately 1.8 times the extent of *Pdgfra^fl/fl^*;*Wnt1-Cre^+/Tg^* cells (0.4885 ± 0.03203 AU, *p* = 0.0357; Figure 10B), while control *Pdgfrb^+/fl^*;*Wnt1-Cre^+/Tg^* cells (0.5897 ± 0.03588 AU) cultured in growth medium had an absorbance reading roughly 2.5 times that of *Pdgfrb^fl/fl^*;*Wnt1-Cre^+/Tg^* cells (0.2394 ± 0.05482 AU, *p* = 0.0018; Figure 10C). Similarly, while there were no significant differences in absorbance readings between control *Pdgfra^+/fl^*;*Wnt1-Cre^+/Tg^* and *Pdgfra^fl/fl^*;*Wnt1-Cre^+/Tg^* cells cultured in starvation medium (Figure 10B), control *Pdgfrb^+/fl^*;*Wnt1-Cre^+/Tg^* cells (0.2047 ± 0.009821 AU) cultured in starvation medium demonstrated a significant increase in absorbance reading over that of *Pdgfrb^fl/fl^*;*Wnt1-Cre^+/Tg^* cells (0.1084 ± 0.01588 AU, *p* = 0.0022; Figure 10C). Taken together, these results confirm the Ki67 immunofluorescence findings and reveal that PDGFRβ plays a more dominant role in proliferation of the facial mesenchyme than PDGFRα past mid-gestation.

## Discussion

Here, we report the first detailed phenotypic characterization of mouse embryos in which both *Pdgfra* and *Pdgfrb* are ablated in the NCC lineage. Our results reveal that the two receptors genetically interact in this lineage during embryogenesis, as phenotypes observed in an allelic series of mutant embryos often worsened with the addition of conditional alleles. We characterized defects in craniofacial development at mid-gestation resulting from combined loss of *Pdgfra* and *Pdgfrb*, including incidences of facial clefting, blebbing, and hemorrhaging. These results confirm the phenotypes we observed from mid-to-late-gestation upon combining the constitutive *Pdgfra^PI3K^* allele together with the conditional *Pdgfrb^fl^* allele and the *Wnt1-Cre* driver (Fantauzzo and Soriano, 2016) and significantly extend those findings by exploring the cellular mechanisms through which these phenotypes arise. The defects observed here were shown to stem from aberrant cNCC migration, as well as reduced proliferation of the facial mesenchyme upon combined decreases in PDGFRα and PDGFRβ signaling. At present, however, our results cannot distinguish between synergistic effects of the two receptors on cNCC activity or whether the observed defects stem from non-redundant roles of PDGFRα and PDGFRβ in this lineage. Importantly, we found that PDGFRα plays a predominant role in cNCC migration while PDGFRβ primarily contributes to proliferation of the facial mesenchyme past mid-gestation.

Our E13.5 gross morphology results further confirm that the *Pdgfra* conditional allele used in this study is hypomorphic, as facial blebbing and facial hemorrhaging were detected at increased incidences in embryos homozygous for this allele in the absence of the *Wnt1-Cre* transgene. While mice heterozygous for a *Pdgfra* null allele are viable (Soriano, 1997), *Pdgfra^fl/−^* embryos are not, exhibiting multiple phenotypes such as spina bifida and cleft palate (Tallquist and Soriano, 2003; McCarthy et al., 2016). Further, *Pdgfra^fl/fl^* mice in our own colony, which are maintained through homozygous intercrosses, generate small litters (average litter size of 4.2 pups at 5–10 days after birth compared to an average of 5.8 pups for wild-type 129S4 litters; *p* = 0.0013) and have shortened snouts with a pigment defect at the facial midline (data not shown). It has been hypothesized that these hypomorphic phenotypes arise due to the presence of a neomycin resistance cassette in the floxed allele that reduces expression of *Pdgfra* (Tallquist and Soriano, 2003). Hypomorphic phenotypes have not previously been attributed to the *Pdgfrb^fl^* allele, and *Pdgfrb^fl/fl^* mice in our colony, which are also maintained through homozygous intercrosses, give birth to litters of expected sizes (average litter size of 6.2 pups at 5–10 days after birth compared to an average of 5.8 pups for wild-type 129S4 litters; *p* = 0.2998).

It is useful to compare and contrast the defects observed in the allelic series of embryos analyzed here with single-homozygous mutant embryos. As mentioned above, a previous analysis found fewer NCCs in PA3–6 in E10.5 *Pdgfra^fl/fl^*;*Wnt1-Cre^+/Tg^* embryos, with bifurcation of the streams entering these arches in a subset of embryos (He and Soriano, 2013). These results are consistent with the findings here, in which *Pdgfra^fl/fl^*;*Pdgfrb^+/fl^*;*Wnt1-Cre^+/Tg^* embryos exhibited cNCC streams with significantly altered sizes, reduced GFP intensity, and noticeable bifurcations compared to the other embryos analyzed. While it is tempting to speculate that these phenotypes stem from defective cNCC directional migration in this context, additional experiments will be required to test this hypothesis. Further, our Ki67 results at E10.5 demonstrated significantly decreased proliferation in the LNPs and MxPs of *Pdgfra^fl/fl^*;*Pdgfrb^+/fl^*;*Wnt1-Cre^+/Tg^* embryos vs. control embryos. This finding is consistent with observed decreases in proliferation in the frontonasal process of E9.5 *Pdgfra^fl/fl^*;*Wnt1-Cre^+/Tg^* embryos as assessed by BrdU staining (He and Soriano, 2013) and in primary MEPM cells derived from *Pdgfra^PI3K/PI3K^* embryos in response to PDGF-AA ligand treatment (He and Soriano, 2013; Fantauzzo and Soriano, 2014), but contrasts with previously-observed decreases in proliferation in the MNPs of E11.5 *Pdgfra^fl/fl^*;*Wnt1-Cre^+/Tg^* embryos (He and Soriano, 2013, 2015). Importantly, our findings are the first to demonstrate a role for PDGFRβ in regulating cNCC migration and proliferation in the developing mouse embryo. While *Pdgfrb^fl/fl^*;*Wnt1-Cre^+/Tg^* embryos do not have cNCC migration defects, the size of cNCC streams entering PA1 and PA2 were significantly longer in *Pdgfra^+/fl^*;*Pdgfrb^fl/fl^*;*Wnt1-Cre^+/Tg^* embryos than double-heterozygous embryos, indicating that PDGFRβ signaling contributes to cNCC migration. However, signaling through this receptor appears to play a more prominent role in facial mesenchyme proliferation, even more so than signaling through PDGFRα. We detected a 37% decrease in cell proliferation in the aPS of E13.5 *Pdgfrb* conditional knock-out embryos compared to heterozygous littermates, which is greater than the decreases in proliferation detected in *Pdgfra* conditional knock-out embryos in the frontonasal process at E9.5 and the MNP at E11.5 (He and Soriano, 2013).

Interestingly, in several parameters examined here, including the lengths of cNCC streams entering the PAs and the percentage of Ki67-positive cells in the LNPs at E10.5, the phenotype of *Pdgfra^fl/fl^*;*Pdgfrb^+/fl^*;*Wnt1-Cre^+/Tg^* embryos was significantly more severe than that of double-homozygous mutant embryos. This result is contrary to our previous observations in which *Pdgfra^PI3K/PI3K^*;*Pdgfrb^+/fl^*;*Wnt1-Cre^+/Tg^* embryos did not exhibit facial clefting at E13.5, while this phenotype was fully penetrant in *Pdgfra^PI3K/PI3K^*;*Pdgfrb^fl/fl^*;*Wnt1-Cre^+/Tg^* embryos (Fantauzzo and Soriano, 2016). The most likely explanation for this finding is that reduced, but not absent, PDGFRβ signaling has a negative effect on cNCC activity and subsequent facial development in a context in which PDGFRα signaling is completely abolished, as observed here. Further studies will be required to determine the mechanism(s) by which this phenomenon occurs.

In *Xenopus*, *pdgfra* is expressed by pre-migratory and migratory cNCCs, while its ligand *pdgfa* is expressed in pre-migratory NCCs and the tissues surrounding migratory NCCs (Bahm et al., 2017). Functional studies revealed dual roles for PDGF-A-dependent PDGFRα signaling in NCC development. During early NCC migration, PI3K/Akt-mediated PDGFRα signaling cell autonomously upregulates N-cadherin to promote contact inhibition of locomotion and cell dispersion. Following initiation of the epithelial-to-mesenchymal transition, migrating NCCs chemotax toward PDGF-A ligand in the surrounding tissue, resulting in directional migration (Bahm et al., 2017). The ligand *pdgfb* is also expressed in tissues adjacent to migrating NCCs in *Xenopus* embryos (Giannetti et al., 2016) and knock-down of this ligand results in impaired cNCC migration and defective development of the craniofacial cartilages and cranial nerves in a subset of morpholino-injected embryos (Corsinovi et al., 2019). In zebrafish, *pdgfra* is similarly expressed by pre-migratory and migratory cNCCs, while its ligand *pdgfaa* is correspondingly expressed at early stages in the midbrain and later in the oral ectoderm (Eberhart et al., 2008). A hypomorphic zebrafish mutant of *pdgfra* exhibits palatal clefting and a shortened neurocrania due to defective cNCC migration (Eberhart et al., 2008; McCarthy et al., 2016). *Pdgfrb* is also expressed by migratory cNCCs in zebrafish, and the phenotypes observed in *pdgfra* mutants are exacerbated in double *pdgfra;pdgfrb* mutant fish in which cNCCs fail to properly condense in the maxillary domain (McCarthy et al., 2016). In contrast to a previous study in which cNCC migration was reportedly unperturbed upon combined ablation of *Pdgfra* and *Pdgfrb* in the murine NCC lineage (Richarte et al., 2007), our results confirm the findings in lower vertebrates that both receptors play a role in NCC migration and that aspects of the phenotype observed upon conditional ablation of *Pdgfra* in the NCC lineage are exacerbated in double-homozygous mutant embryos.

In summary, our findings provide insight into the distinct mechanisms by which PDGFRα and PDGFRβ signaling regulate cNCC activity and subsequent craniofacial development in a mammalian system. Future studies will seek to identify the intracellular signaling molecules and gene expression responses that mediate the effects of these receptors on migration and proliferation.

## Data Availability Statement

The original contributions presented in the study are included in the article. Further inquiries can be directed to the corresponding author.

## Ethics Statement

The animal study was reviewed and approved by The Institutional Animal Care and Use Committee of the University of Colorado Anschutz Medical Campus.

## Author Contributions

KF conceived and designed the study. JM, RL, and KF performed experimentation. JM and KF analyzed data. KF wrote the original draft of the manuscript, which was revised and edited in an iterative process with JM. All authors contributed to the article and approved the submitted version.

### Conflict of Interest

The authors declare that the research was conducted in the absence of any commercial or financial relationships that could be construed as a potential conflict of interest.
